# Long COVID: The evolution of household welfare in developing countries during the pandemic

**DOI:** 10.1016/j.worlddev.2023.106485

**Published:** 2024-03

**Authors:** Ben Brunckhorst, Alexandru Cojocaru, Yeon Soo Kim, Maurice Kugler

**Affiliations:** a Poverty and Equity Global Practice, World Bank Group, United States; b Schar School of Policy and Government, George Mason University, United States; c Interdisciplinary Center for Economic Science, George Mason University, United States; d Center for Microeconomic Policy Research, George Mason University, United States

**Keywords:** COVID-19, Welfare, Inequality, Labor market, Phone surveys

## Abstract

This study examines household welfare dynamics during the COVID-19 pandemic, using harmonized data from over 300 phone surveys in 80 countries during 2020 and 2021, representing more than 2.5 billion people. The analysis traces out the evolution of employment and income across and within countries as restrictions on economic activity were relaxed. We show some groups initially experiencing higher rates of employment loss – including women, informal workers, and those with less education – also recovered jobs at a slower pace. Based on panel regressions, changes in policy stringency were associated with unequal employment outcomes. Labor market transitions were toward jobs of inferior quality on average, especially for workers with less education. Household income dynamics suggest uneven impacts in the intensive margin of employment consistent with these transitions. Lower wages were not offset by additional social assistance. Taken together, these dynamics may amplify the inequality impacts of the pandemic over the medium to long term.

## Highlights

• We use 343 phone surveys from 80 countries to provide new evidence on household welfare dynamics in developing countries during COVID-19.
• After losing jobs at higher rates, workers with low levels of education and women were also less likely to recover employment into 2021.
• The relationship between policy stringency and employment over time varied by location, gender and education level.
• Labor market transitions were toward jobs with lower returns in agriculture and self-employment, especially in poorer countries.
• Household income dynamics suggest employment impacts were not offset by additional social assistance.

## 1 Introduction

Three years into the COVID-19 pandemic, the world was still recovering from disruptions to lives and welfare that are unprecedented in recent times. In 2020, economic activity contracted in nine out of ten economies – worse than during the Great Depression of the 1930s, World War II or the global financial crisis of 2007–08 – while global poverty increased for the first time since 1998, adding 70 million to the number of extreme poor (World Bank, 2022a). Labor market impacts have been particularly severe with workplace disruptions due to social distancing requirements and global supply chain disruptions affecting many sectors and occupations. Recovery has been slow and challenging, impeded by inflationary pressures and the war in Ukraine.

Early analysis of household phone surveys – one of the few available sources of data during pandemic lockdowns – suggested that the impact of COVID-19 on employment and incomes was unequal across and within developing countries. Employment and incomes dropped sharply almost everywhere but particularly in middle-income countries (MICs) during the first few months of the pandemic when governments adopted sweeping containment measures, including lockdowns, quarantines and school closures. Within countries, restrictions on mobility and economic activity were associated with larger losses in employment and income among more vulnerable population groups, including those with lower levels of education, women, and urban informal workers (Bundervoet et al., 2022, Kugler et al., 2023).

This study updates existing knowledge of the employment and income impacts of the pandemic, using household level data from a considerably larger sample of developing countries, and describes how welfare evolved during the economic crisis throughout 2020 and 2021. We use harmonized data from high-frequency phone surveys (HFPS) in 80 countries from five out of six World Bank regions, representing a combined population of over 2.5 billion.^1^ The HFPS were collected between April 2020 and December 2021. Most countries represented by the data conducted at least four survey rounds, recontacting the same households. While the data is unique in its coverage, especially during a period when traditional household surveys were at least temporarily halted in many countries, it comes with a set of important caveats. For example, previous studies highlight measurement challenges associated with phone survey data collection and biases that threaten representativeness (Gourlay et al., 2021). This paper considers these issues and additional caveats related to the way HFPS measure employment, which have received less attention but are important for interpretation of the evidence.

One less known aspect of the COVID-19 pandemic, and the focus of this paper, is potential scarring effects and their implications for poverty and inequality in the medium term. We show that extensive work stoppages peaking in the second quarter of 2020 persisted, to some degree, through 2020 and in most countries all the way through 2021. We confirm earlier findings that female workers and those with lower levels of education experienced more severe employment losses on account of the pandemic disproportionately impacting services sectors in which they tend to work, and non-wage types of employment. The uneven distribution of care responsibilities left women assuming the bulk of the increased needs of care for children following school closures. We also find women, especially those with children and lower levels of education, have experienced smaller gains in employment through the second half of 2020 and 2021 following the initial shock. These unequalizing dynamics are partially attributed to the differential effects of containment policies by employing individual level panel regression models combined with data capturing the variation in policy stringency over time.

While there is evidence of work restarting after initial job losses, one prominent feature of the recovery is the increased prevalence of self-employment relative to before the pandemic. It appears that the mass job displacement that was caused by the COVID-19 labor market shock was buffered partially by informal employment. Given that informal self-employment in developing economies tends to be concentrated in low-return activities, this suggests that while employment rates improve, new jobs may be of lower quality than those held prior to the pandemic. Similarly, participation in the agriculture sector in low-income countries (LICs) and lower middle-income countries (LMICs) increased significantly.^2^

Household income dynamics indicate changes in the intensive margin of employment consistent with transitions to work with lower returns, and these losses were not offset by social assistance. Indeed, reported income losses remained widespread into 2021 in many countries. Most governments implemented COVID-19 fiscal packages to counter the pandemic impacts on the economy and people, with higher-income countries spending significantly more than lower-income countries on support to households and firms. A synthesis of microsimulation studies suggests that increases in poverty were not mitigated by social protection in poorer countries (World Bank, 2022a). Phone surveys show that benefit coverage expanded over the course of the crisis, but not sufficiently to reach many households losing income. Households adopted harmful coping strategies, including the sale of assets, which could hurt their future productive capacity and lead to higher poverty and inequality in the future.

The remainder of this paper is structured as follows. Section 2 briefly reviews existing studies on the welfare impacts of the COVID-19 pandemic and describes the main contributions of this study. Section 3 describes the data used in the analysis, defines the main variables used to monitor employment and welfare impacts, and discusses some of the data-related caveats. Section 4 presents the main findings related to the patterns of employment losses and subsequent recovery across different countries and population groups. Section 5 looks at household income dynamics and reports evidence of welfare impacts due to changes in the intensive margin of employment which could reinforce inequality in the longer-term if the losses are left unmitigated. Section 6 concludes.

## 2 Welfare impacts of COVID-19: Existing evidence and knowledge gaps

A number of studies have documented the multifaceted welfare impacts of the COVID-19 pandemic both across and within countries, focusing primarily on employment and income losses. These resulted from restrictions to mobility and economic activity that were imposed by governments in order to contain the spread of the coronavirus as well as the ensuing impact of global demand and supply shocks.^3^ In developing countries, analyses often rely on phone surveys including the World Bank’s HFPS, since the collection of regular Household Budget Surveys or Labor Force Surveys (LFS) was largely halted during 2020–2021. HFPS data revealed high rates of work stoppages early in the pandemic, particularly in the industry and services sectors, lower wages for work performed, as well as income losses at the household level that correlate with the stringency of containment policies (Bundervoet et al., 2022, Khamis et al., 2021, Kugler et al., 2023). For instance, Bundervoet et al. (2022) find, based on a sample of 31 countries, that more than a third of respondents reported having stopped working since the onset of the pandemic, and almost two-thirds of households reported a decrease in total income. These job and income losses were associated with significantly higher levels of food insecurity at the household level.

The early analyses conclude that the welfare impacts precipitated by the economic crisis were not evenly distributed either across or within countries. Across countries, employment and incomes dropped sharply almost everywhere, and nearly all LICs and LMICs saw poverty increase in 2020. Globally, the pandemic led to a broad shock across the global income distribution, though households with per-capita incomes above $20 per day (in 2017 PPP) were much less affected than the rest (World Bank, 2022a).

Within countries, evidence suggests the brunt of the burden from the COVID-19 labor market shock was borne by women, less educated, and urban workers (Bundervoet et al., 2022, Kugler et al., 2023). The disproportionate impact on these population groups has also been corroborated in a number of countries that continued standard survey data collection, including Türkiye (Aldan et al., 2021), South Africa (Köhler et al., 2023), Brazil (World Bank, 2022b), Vietnam (Dang et al., 2023), and India (Deshpande, 2020). Similar patterns are also observed in advanced economies. For instance, harmonized data from the EU Statistics on Income and Living Conditions (EU-SILC) show that in Q2 of 2020 the risk of temporary layoffs or reduced hours declines with household income. Chetty et al. (2020), based on data from the United States, find that employment losses between January and April 2020 decreased monotonically with income, from 37 percent in the bottom wage quartile to 14 percent in the top wage quartile.

These differential impacts across population groups are in part because the ability of workers to continue jobs remotely is higher among better-educated workers due to the digital divide and the cognitive nature of work for high-skill occupations. In the case of developing countries, some studies have analyzed task characteristics across occupations to estimate the share of jobs that can be done at home. Dingel and Neiman (2020) find the share to be increasing with country income levels, with fewer than 25 percent of jobs in Mexico and Türkiye being amenable to working from home. World Bank (2020) uses data from India to estimate that less than 10 percent of jobs are amenable to working from home at the 70th and lower percentiles of the earnings distribution and suggests that the actual rate is likely much lower because of constraints to accessing digital technology. Aminjonov et al. (2023) show visits to workplaces dropped less in poorer regions using subnational mobility data. Gottlieb et al. (2021) find that just 13.3 percent of urban workers in Brazil and 10.6 percent in Costa Rica were actually working from home during the pandemic. This suggests that the ability to work from home likely played a much more diminished role in developing country contexts. In addition to the feasibility of home-based work, there is also considerable evidence of the gender gap in the care responsibilities precipitated by the pandemic and accompanying school closures which may have led to a reduction in women’s labor force participation (Albanesi and Kim, 2021, Bau et al., 2022, Lee et al., 2021, Levine et al., 2021, Miguel and Mobarak, 2022).^4^

Beyond the initial impact, there is some evidence that those who were more likely to initially experience losses – women, urban workers, and the low-educated – also recovered more slowly during the initial relaxation of containment measures in mid-2020 (Narayan et al., 2022). ILO (2021) finds that the labor market recovery stalled during 2021 based on data from LFS, with little progress since the final quarter of 2020. Kim et al. (2021) using phone survey data from East Asian countries, find that employment impacts were widespread across the income distribution when mobility restrictions were stringent, but it was more difficult for poorer workers to regain employment once restrictions were eased. This pattern is also found in the US (Chetty et al., 2020, Lee et al., 2021), Colombia (Alvarez & Pizzinelli, 2021), and India (Kugler & Sinha, 2020).

Against this background, our paper’s first contribution is the use of a unique data source to describe the evolution of employment and income impacts during the first two years of the pandemic, across and within developing countries. The HFPS are, to our knowledge, the most comprehensive household data collection effort during the pandemic or any preceding global crisis. By drawing on the full global and temporal extent of this dataset, we greatly expand existing knowledge of the impacts of the pandemic in developing countries (Miguel & Mobarak, 2022).

Secondly, we apply panel econometric models to more clearly identify characteristics associated with loss and recovery of both employment and income within countries. Since our data tracks respondent level employment outcomes over time as containment measures became more and less restrictive depending on contagion risk, we are able to specify a respondent fixed effects model that better isolates the effects of pandemic specific features of the COVID-19 shock across individuals using a large sample of developing countries.

Thirdly, our study uncovers hidden labor market impacts, beyond adjustments at the extensive margin. Focusing solely on levels of employment could obscure some of the welfare effects of the pandemic. For example, transitions into agriculture and self-employment would likely be associated with lower quality informal jobs, yielding lower remuneration vis-à-vis prepandemic wage employment. Such transitions may be more prevalent among lower-income households, who lack access to safety nets and cannot sustain prolonged unemployment spells for fear of not being able to cover basic necessities. We analyze employment transitions during the pandemic, revealing changes in the quality of employment across different population groups.

**Table 1:** Sample composition across three periods of analysis.

	Number of countries with HFPS				Share of population (%)	Survey waves
	Apr-Jun20	Jul-Dec20	2021	Total		
High income	5	5	7	8	7	26
Upper-middle income	14	18	24	26	36	90
Lower-middle income	19	19	17	26	30	112
Low income	13	18	12	20	85	115
						
East Asia and Pacific	7	8	10	11	29	40
Europe and Central Asia	5	9	9	9	15	77
Latin America and Caribbean	14	14	24	24	94	66
Middle East and North Africa	5	5	2	7	38	26
Sub-Saharan Africa	20	24	15	29	84	134
						
Fragile and Conflict-Affected	13	14	9	19	37	62
						
**Total**	**51**	**60**	**60**	**80**	**33**	**343**

*Notes*: Surveys are attributed to a period based on the mean month of data collection. Most surveys were conducted within two calendar months.

Finally, this study analyses household income dynamics in light of employment impacts, which indicates the extent of unrecovered welfare losses over the pandemic. We show evidence of unequal welfare impacts consistent with changes in the intensive margin of employment suggested by labor market transitions, and show these losses were not fully compensated by income from other sources, such as pandemic era public assistance.

Despite the uneven impact of the pandemic across population groups, the estimated short run impacts of COVID-19 on aggregate within-country income inequality so far appear to be mixed (World Bank, 2022a).^5^ However, studies of previous pandemics suggests their impact on inequality can peak 4–5 years after the onset of the pandemic (Furceri et al., 2022). The impacts of the COVID-19 pandemic on inequality could worsen over time if the scarring effects of sustained employment and income losses as well as distress asset sales and learning disruptions for children in poorer households hinder recovery relative to better off households over the medium-term (Kim et al., 2021, Neidhöfer et al., 2021, World Bank, 2022a).

## 3 Data and methods

**Fig. 1:**
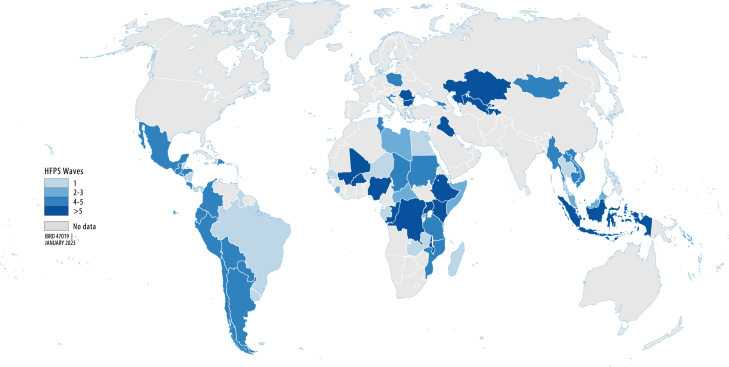
Number of HFPS waves across countries.

The main data used in this study was from the November 2022 vintage of the World Bank’s HFPS dataset, collected to monitor the impact of the pandemic on households. The first surveys were conducted in April 2020, with data collection continuing into 2022 in some economies. Since questionnaires differed by country and across survey waves, responses were harmonized to construct a globally comparable dataset with up to half a million observations for a given indicator. Data used for analysis in this paper were collected from 80 countries in five of the six World Bank regions over 343 survey waves between April 2020 and December 2021. The breakdown of countries with harmonized survey data by income level, region and timing is shown in Table 1.^6^ More than half are LICs and LMICs, including 19 Fragile and Conflict-Affected Situations (FCS). Of the 80 countries with surveys, 53 are located in Latin America and the Caribbean (LAC) and Sub-Saharan Africa (SSA), such that averages reported are strongly influenced by these regions (Fig. 1). The countries represented in the harmonized database cover 85 percent of the population in LICs, and over 80 percent of the population in both LAC and SSA. Previous papers using the HFPS describe the harmonization process in more detail (Bundervoet et al., 2022, Khamis et al., 2021, Kugler et al., 2023).

Phone surveys have the advantage of collecting data widely and rapidly. They were the only option in many developing countries early in the COVID-19 crisis when nearly all face-to-face surveys ceased. However, there are important limitations to be considered. First, they can only draw a sample from the phone-owning population, such that households with limited or no access to phones will be underrepresented. Second, response rates are often quite low compared to in-person interviews and attempts to address nonresponse bias will be imperfect. Third, the scope and type of questions that can be asked is often limited compared to in-person surveys in order to keep them short and to minimize refusal.

Two main sampling strategies were used for the World Bank COVID-19 phone surveys, with implications for the populations they were intended to represent. Surveys in 36 countries, especially those in LAC, used pure Random Digit Dialing (RDD) or assisted RDD. This method samples from all active landline and mobile phone numbers, such that RDD surveys would be representative of the population (over 18 years) with an active phone number if survey completion and response rates were perfect. On the other hand, surveys in 31 countries used a subsample drawn from a prepandemic nationally representative survey. The latter often collected the contact details of the household head, meaning that phone surveys using this sampling frame tend to overrepresent household heads and underrepresent members who are neither heads nor spouses (Gourlay et al., 2021). This means that individual level indicators such as employment outcomes may be biased due to respondent selection within households. The remainder of surveys use other pre-existing lists to identify the sampling frame.

To help correct for the non-representativeness of surveyed households, household sampling weights were constructed. These adjust for differential response rates among subgroups of the population, with the objective of obtaining estimates as close to nationally representative as possible. Addressing the non-representative selection of individuals poses a greater challenge to generating statistics that are representative of individual level outcomes such as engagement in the labor market. Kugler et al. (2023) examine this source of bias for five countries where surveys collected employment information for all household members and find that phone surveys overstate employment rates for the full population even once sampling weights are used. Respondents are typically more likely to be employed than the population average. Encouragingly, their analysis suggests that phone surveys do reasonably well at tracking disparities across gender, education, and urban/rural groups, including trends over time. However, they find significant sample bias for age group comparisons, which are therefore not reported in this paper.

We use household sampling weights for all our analysis, including employment outcomes, such that statistics should be interpreted as the share of households with a respondent having a particular employment outcome.^7^ Countries are weighted equally so that summary statistics are calculated as averages of country averages, intended to represent a global picture of country experiences rather than the story of global population aggregates. The same approach to weighting is used for all regression analysis.

**Fig. 2:**
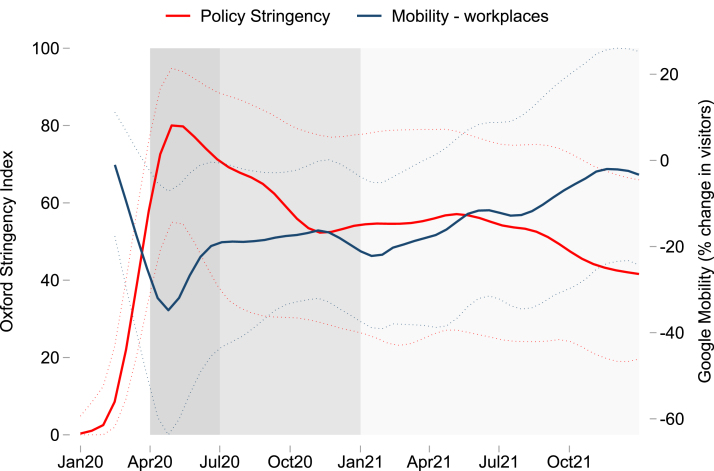
Policy stringency and mobility trends in economies studied. *Notes*: The shaded regions represent the three time periods used for descriptive analysis. Solid lines represent the median of each indicator and dotted lines show the 10th and 90th percentiles. The OxCGRT stringency index is available for 76 out of 80 countries with HFPS data. Google Community Mobility data is available for 57 countries with HFPS data.

To keep the analysis tractable, we present descriptive statistics for three time periods broadly based on different stages of the pandemic, as shown in Fig. 2. The first period from April 2020 to June 2020 represents the initial impact of the pandemic, marked by strict containment policies in many countries including lockdowns and school closures, as captured by the Oxford Covid-19 Government Response Tracker (OxCGRT) stringency index (Hale et al., 2021).^8^ This corresponds with a substantial decrease in visits to workplaces and transit stations, while people spent more time in residential areas, according to Google COVID-19 Community Mobility Reports (Google LLC, 2022). The second period, July 2020 to December 2020, is characterized by a gradual withdrawal of some containment measures and increased mobility following the initial shock, though there are regional differences. The third period covering 2021 is distinguished by stalled progress in many countries, with little change in policy stringency on aggregate, but some return to normal levels of mobility. More significant differences emerge between regions and countries in this third period (Figure A.2).

When countries fielded multiple surveys within one of these time periods, we select a single survey to calculate summary statistics for each period (Figure A.2). For the initial period, we choose the survey month corresponding with the highest stringency index in order to estimate peak welfare impacts at the onset of the pandemic. To look at subsequent recovery in welfare and employment outcomes, we use the survey month corresponding to the lowest stringency index in the second half of 2020, and the most recent survey collected in 2021 for the third period.^9^

Google COVID-19 Community Mobility Reports and Oxford COVID-19 Government Response Tracker.

For employment outcomes, the sample is restricted to households in which the respondent was between 18 and 64 years old, since employment questions were only asked of the respondent. Survey waves were only included in the calculation of summary statistics if the indicator had a response rate of more than 50 percent. Table 2 describes the main welfare outcomes of interest.

While HFPS data allow us to compute estimates of the share of employed adults both prepandemic (based on recall) and in each subsequent HFPS round, comparisons between employment levels before and after the onset of the pandemic are made difficult by the fact that current and prepandemic employment are queried differently, and are thus not comparable. To minimize the bias this introduces in inference, the analysis of employment losses in Section 4 primarily compares changes in employment (losses and gains), computed in terms of differences between employment status reported by the respondents in each survey round and their prepandemic recall, over time. If recall bias is constant across survey rounds, these comparisons are internally valid because errors associated with recall bias are cancelled out. Since prepandemic employment was queried in multiple survey rounds, we use an individual’s earliest response and treat it as time invariant in our analysis by matching respondents across panel survey rounds where possible. This both minimizes the recall period and reduces potential variation in recall bias across survey rounds. We then confirm that prepandemic employment estimates are stable across survey rounds (Figure A.3), implying the constant recall bias assumption is valid in our data.

**Table 2:** Harmonized welfare indicators.

Topic	Indicator	Countries	N
Employment	Respondent was working at the time of the survey	77	471,967
	Respondent had stopped/started working at the time of the survey relative to prepandemic	75	443,902
	Respondent changed job, sector or employment type relative to prepandemic, if working before the pandemic and at the time of survey	Job: 69 Sector: 54 Type: 43	184,324 115,828 96,152
	Respondent gained employment during the pandemic, if reported not working in a previous HFPS wave (panel respondents)	52	136,478
	Respondent recovered employment during the pandemic, if reported stopped working in a previous HFPS wave relative to prepandemic (panel respondents)	52	78,019

Income	Household total income decreased relative to the prepandemic reference period	54	186,073
	Household received no or partial payment for wage work in the past week, if the respondent was a wage worker	47	36,421
	Household gained income during the pandemic, relative to the previous HFPS wave	29	102,091
	Household recovered income during the pandemic, if reported income loss in a previous HFPS wave relative to prepandemic (panel households)	23	56,360

Assistance	Household received any form of public assistance since the start of the pandemic	70	359,736

*Notes*: The number of observations includes all surveys; questionnaires varied across survey rounds and countries.

A further cross-check of employment estimates from HFPS can be provided by comparisons with labor force survey (LFS) data, primarily in UMICs and high-income countries (HICs) that continued to collect LFS during the pandemic after initial interruptions. It should be noted that employment estimates from the two sources are not strictly comparable, and any inconsistencies could be the result of differences in the number, sequence and wording of questions, differences in the timing of surveys, and important distinctions between the populations represented as well as the aforementioned recall bias.^10^ The comparison suggests that the HFPS and LFS capture similar employment dynamics in countries with overlapping data, although there are differences in magnitude of losses and gains that the two data sources capture. The share of respondents currently working in the HFPS is strongly correlated with trends in the employment-to-population ratio reported in the ILO database (R${}^{2}$ = 0.869, Figure A.4). Discrepancies in terms of how closely employment trends mirror each other at the country level tend to be less consequential in country or population group aggregates, which is the level of analysis in this paper. We find employment gaps by location, gender, and employment type display similar trends across the two sources of data in LAC, even though they are each subject to measurement error (Figure A.5). Since LFS data is available for very few lower income countries, the HFPS remain one of the only sources of household level data from the pandemic era that track employment across many developing countries.

## 4 Employment dynamics during the pandemic

This section uses the most comprehensive and up-to-date cross-country data available to trace the evolution of employment, work stoppages and acquisition of new jobs throughout 2020 and 2021, and examine transitions across sectors and types of employment. There are several ways through which labor market outcomes could be affected during the crisis. These include outright job losses and gains, as well as changes in the intensive margin such as variation in wages or working hours including those related to employment transitions. Some workers may also be temporarily absent from their job; job seekers may face worse job prospects while having to reduce their job search efforts due to the pandemic; some may decide to continue education instead. While plausible, these latter mechanisms are difficult to observe with the data at hand. Instead, this study is restricted to focusing mainly on estimates of outright job losses and gains over time and across different groups, as well as some, though not all, changes in jobs.

### 4.1 What are the main patterns of initial employment losses and subsequent employment gains?

**Fig. 3:**
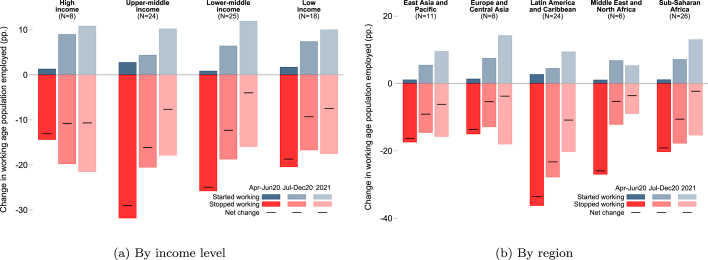
Employment dynamics across country groups. *Notes*: The figure shows changes in employment as a share of the working age population relative to prepandemic, decomposed into job losses and gains, by income level in panel (a) and by region in panel (b). Estimates are based on a sample of 246,618 respondents from 75 countries that have a survey collecting sufficient employment information in any of the three periods that we are reporting on. Household sample weights are used within countries and countries are weighted equally.

Fig. 3 shows the evolution of employment by country income group and region over three periods, based on the HFPS.^11^ Changes in employment are decomposed into changes in the share of the working age population starting work relative to the prepandemic reference period (gains, if they were unemployed prepandemic), and changes in the share stopping work (losses, if they were working prepandemic). The net change in employment is the sum of these two adjustments. This decomposition shows that while net changes in employment from April-June 2020 were almost entirely driven by sudden job losses, during the second half of 2020 and especially in 2021, the employment recovery was driven not only by relatively lower rates of job losses, but also by a higher share of previously unemployed adults starting work.

Consistent with earlier studies, the updated HFPS dataset confirms widespread initial labor market losses. Overall, the share of the working age population who reported being employed in April-June 2020 was, on average, 31 percent lower relative to prepandemic levels (Table 3). However, as we note in the previous section, the estimated magnitude of these initial job losses, inclusively in the earlier studies, should be interpreted with caution, as the size of any recall bias associated with the framing of the prepandemic employment question in the HFPS is unknown.^12^

COVID-19 High-Frequency Phone Surveys.

Given that many countries did not have a survey in all three periods, it is possible that dynamics of certain outcomes observed over time may driven by changes in the sample composition. To address this concern, we also consider employment estimates for a sample restricted to countries with data points in all three time periods. We report results for this “country panel” in Table A.1. Overall, employment trends for a balanced panel of 31 countries are similar to results for the full sample.

Updated estimates reaffirm that initial labor market impacts, as well as the pace of the subsequent gains, varied considerably across country groups. MICs experienced the largest employment losses in the initial stage of the pandemic, followed by an increase in employment levels in the second half of 2020 and through 2021. Among a small set of HICs, the initial magnitude of job losses was much smaller, but has been followed by a slower pace of recovery. In LICs, employment losses were subdued compared to those reported in MICs. There could be several contributing factors. First, the extent of restrictions to mobility and economic activity, as measured by the OxCGRT stringency index, was somewhat lower on average in LICs. In addition, LICs have a much higher share of agricultural employment and a higher share of population residing in rural areas, which have not been affected as much by mobility restrictions as those living in urban areas. Also, the HFPS data do not directly capture changes in the intensive margin, such as changes in hours worked which are shown to be significant (ILO, 2021). This may explain why a much larger share of households in LICs report to have lost income since the pandemic started. Employment recovery among LICs appears to stall in 2021, with gains driven only by new entrants. Unlike in MICs, the share of adults that had stopped working compared to before the pandemic did not continue to fall between the second half of 2020 and 2021.

Across regions, employment fell most in the early phase of the pandemic and employment loss was substantially larger in LAC compared to other regions. Employment levels recovered during the second half of 2020, most significantly in Europe and Central Asia (ECA), LAC and SSA, relative to prepandemic levels. The EAP region diverges from these patterns when considering results in a panel of five countries, which indicate limited recovery from initial labor market losses throughout 2020 and into 2021. Results for a smaller but stable sample of countries also suggest little recovery in employment levels during 2021 in ECA and SSA (Table A.1).

### 4.2 All in this together? employment gains across different population groups

One of the main findings in studies of initial COVID-19 impacts described in Section 2 related to the uneven impacts of the pandemic on different population groups. Those with lower levels of education, informal employment, and women have been impacted to a greater degree initially by job losses. Table 3 reports relative employment losses across population groups in 2020 and 2021, as a percentage of each group’s prepandemic employment level. Our main interest is in comparing the magnitudes of relative employment changes across different population groups over time. To make comparisons across countries in terms of education level, we define low education as primary or less in LICs and LMICs, and secondary or less in UMICs and HICs.

The estimates from the full HFPS sample confirm that the initial impacts of the pandemic on employment were more pronounced for women, urban workers, and those with lower levels of education. There was proportionally greater employment loss among women relative to prepandemic levels, and this gap persists into 2021. Work stoppages – the main component of net employment changes – decreased over time, but the rate of returning to employment tended to be lower among women, who report a higher incidence of work stoppages in the later part of the pandemic. For a number of countries, particularly in the LAC region, women experienced larger declines in employment during the initial phase of the pandemic, and subsequent gains in employment either proceed at roughly the same pace among women and men, or gender disparities actually widened over time (Figure A.6).

**Table 3:** Relative employment losses across population groups over time.

	Employment loss relative				
	to prepandemic level (%)			Countries	N
	Apr-Jun20	Jul-Dec20	2021		
All countries	30.6	16.8	8.2	75	246,618
					
Female	33.6	20.1	10.0	73	103,900
Male	27.2	14.6	7.4	73	139,142
**Female/Male**	**1.24**	**1.38**	**1.35**		
					
Rural	28.7	15.1	7.5	67	92,426
Urban	31.5	17.3	9.3	69	135,127
**Rural/Urban**	**0.91**	**0.87**	**0.81**		
					
Low education	33.6	19.4	8.1	59	92,320
High education	29.1	17.8	7.9	59	89,509
**Low/High**	**1.15**	**1.09**	**1.03**		

Employment also fell proportionately more among those with lower levels of education in April-June 2020, following the onset of the pandemic. Relative to prepandemic employment levels, this gap appears to close in subsequent HFPS waves, in the full sample and in the country panel (Table A.1). However, this is driven by previously unemployed workers in the low education group starting jobs at a faster rate, rather than those that stopped working returning to jobs more quickly.

To get a cleaner picture of the characteristics associated with job losses and subsequent employment recovery, we present results from regressions that make use of the panel structure of the data. The basic specification for our linear probability model is given by:

(1) $$Y_{i,t}=\alpha +\beta_{n}X_{i}+\gamma Z_{c,t}+\delta_{c}+\epsilon_{i,t}$$

where $Y_{i,t}$ is the outcome of interest for household i at time t (stopped working or recovered employment) and $X_{i}$ is a vector of time invariant respondent and household characteristics influencing employment outcomes during the COVID-19 shock, including household location, gender, education level, employment sector, and whether there were children in the household (or the union of such characteristics). $Z_{c,t}$ is OxCGRT stringency index in country c at time t (standardized within each country), $\delta_{c}$ is a country fixed effects term, controlling for all observed and unobserved time invariant factors at country level, and ϵ is a mean zero error term. Specification (1) estimates the average within-country effect of each characteristic on the conditional probability of the outcome of interest, given by β.

**Fig. 4:**
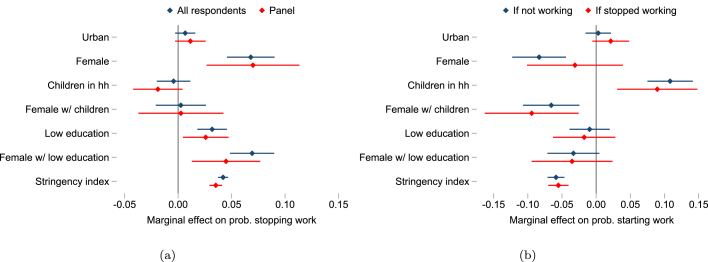
Characteristics associated with employment loss and recovery. *Notes*: The figure visualizes results in Table 4. It shows the marginal effect of each variable on the probability of employment loss relative to prepandemic in panel (a), and of employment gain during the pandemic in panel (b). 95% confidence intervals shown.

COVID-19 High-Frequency Phone Surveys.

Results from these regressions are reported in Table 4.^13^ Fig. 4(a) visualizes these results, showing that those working before the pandemic were significantly more likely to stop working within each country if they were women, lower educated, and during a period of higher policy stringency. Workers in urban places were also marginally more likely to stop work.

We draw on a subsample of almost 40 countries with panel surveys to construct an employment recovery outcome that allows us to use specification (1) to predict the probability that those who initially stopped working subsequently returned to work (as reported in a follow-up HFPS wave) for people with different characteristics. Fig. 4(b) shows women were also less likely to gain or recover employment during the pandemic period, especially in households with children. On average, those with lower education were also less likely to recover employment after stopping work, though the effect is not statistically significant when looking at each gender separately.

Differences in the probability of recovering employment across characteristics are substantial. For example, less educated women with children were, on average, 14–18 percentage points more likely to stop working and 18–19 percentage points less likely to return to work, relative to more educated men with children (Table 4). Taken together, the results imply a widening gender and education employment gap over time, accentuated by the burden of childcare on women. We find that those working in agriculture before the pandemic were seven percentage points less likely to stop working than those in other sectors, although there are no statistically significant differences in employment recovery outcomes across sectors during the pandemic (controlling for other characteristics). The different employment transitions observed in HFPS data, and their welfare implications are discussed in more detail in Section 4.3. Lower rates of employment recovery were also observed during periods of more stringent containment policies.

**Table 4:** Characteristics associated with employment loss and recovery during the pandemic.

	Stopped working				Started working during pandemic		
	All respondents		Panel		If not working	If stopped working	
	(1)	(2)	(3)	(4)	(5)	(6)	(7)

Urban	0.007	0.000	0.011	0.009	0.003	0.021	−0.000
	(0.005)	(0.007)	(0.007)	(0.010)	(0.010)	(0.014)	(0.020)
Female	0.068***	0.065***	0.070***	0.051*	−0.083***	−0.031	0.003
	(0.011)	(0.018)	(0.022)	(0.029)	(0.020)	(0.036)	(0.043)
Children	−0.004	−0.021**	−0.019	−0.038***	0.108***	0.089***	0.144***
	(0.008)	(0.010)	(0.012)	(0.015)	(0.017)	(0.030)	(0.040)
Female × Children	0.003	0.006	0.003	0.021	−0.066***	−0.094***	−0.131***
	(0.012)	(0.017)	(0.020)	(0.027)	(0.021)	(0.035)	(0.044)
Low education	0.032***	0.037***	0.026**	0.032**	−0.010	−0.018	−0.038
	(0.007)	(0.009)	(0.011)	(0.014)	(0.015)	(0.023)	(0.027)
Female × Low edu.	0.069***	0.076***	0.045***	0.060***	−0.033*	−0.035	−0.023
	(0.011)	(0.015)	(0.016)	(0.022)	(0.019)	(0.030)	(0.034)
Stringency index	0.042***	0.048***	0.035***	0.044***	−0.059***	−0.055***	−0.066***
	(0.002)	(0.003)	(0.003)	(0.004)	(0.006)	(0.008)	(0.007)
Mining/Manufact.		0.077***		0.076***			0.009
		(0.015)		(0.023)			(0.040)
Commerce		0.068***		0.067**			0.002
		(0.018)		(0.027)			(0.030)
Other services		0.067***		0.052***			0.025
		(0.013)		(0.020)			(0.028)

Country FE	✓	✓	✓	✓	✓	✓	✓
R${}^{2}$	0.08	0.09	0.09	0.09	0.15	0.11	0.09
N	198,487	146,364	148,938	106,394	72,723	41,752	31,321
Countries	52	46	37	31	36	36	30

*Notes*: The table reports results from a linear probability model with country fixed effects. Household sample weights are used within countries and countries are weighted equally. The OxCGRT stringency index is matched to surveys at month level and standardized within countries across the period April 2020 to December 2021. The baseline sector (not shown) is agriculture. Standard errors are robust. * p<0.1, ** p<0.05, *** p<0.01.

While specification (1) demonstrates employment impacts during the pandemic were uneven across population groups within countries, it is possible that the estimates are picking up not only COVID-19 specific impacts, but also other labor market and economic conditions occurring during this period which may be unrelated to the pandemic. While pandemic related effects are generally expected dominate other factors during our period of analysis ending before the war in Ukraine, we explore more specifically the heterogeneous experience of workers during COVID by interacting household characteristics with the time-varying stringency index as follows:

(2) $$Y_{i,t}=\alpha +\gamma Z_{c,t}+\beta_{n}X_{i}\times Z_{c,t}+\delta_{i}+\epsilon_{i,t}$$

Since HFPS data tracks welfare for the same respondent, specification (2) allows us to estimate how an outcome of interest Y varies with changes in policy stringency Z across workers with different characteristics X, controlling for all time-invariant characteristics at respondent level ($\delta_{i}$). γ estimates the average within-person effect of a one standard deviation increase in the country level stringency index on the probability of employment loss and recovery, while β estimates heterogeneous effects by location, gender and education level.

While the inclusion of individual-level fixed effects allows us to account for time-invariant confounders such as, for instance, differential sorting within countries of low-skilled workers into sectors or occupations that are generally more or less prone to turnover, it is still the case that changes in containment policies, summarized by the stringency index, are correlated with other important time-varying features of the pandemic. For example, the global demand shock affected local labor demand and supply in specific sectors when restrictions around the world were being implemented at the same time (Brinca et al., 2021). The public health crisis and risk of contagion affected some occupations and individual employment decisions, and was simultaneously a key determinant of policy decisions (del Rio-Chanona et al., 2020). Income support in poorer regions was associated with a decrease in workplace mobility during lockdown periods, but the reason for expanding safety nets was in large part to alleviate the impact of containment measures (Aminjonov et al., 2023). As such, specification (2) can capture the combined effect of these developments to the extent they are correlated with changes in policy stringency over time. Results are reported in Table 5. A one standard deviation increase in the stringency index is associated with an increase in the probability of stopping work by three percentage points, on average, and a decrease in the probability of subsequently recovering employment by seven percentage points.

**Fig. 5:**
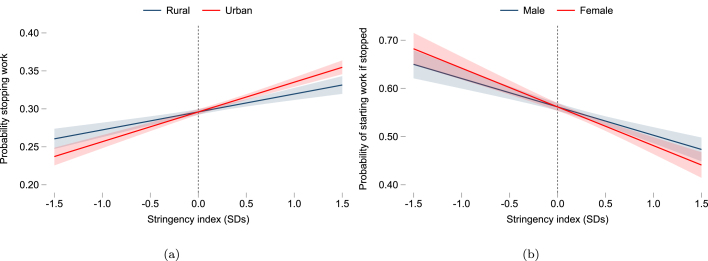
Effect of policy stringency on employment loss and recovery from panel regressions. *Notes*: The figure visualizes results in Table 5. It shows the predicted probability of employment loss as a function of policy stringency by location in panel (a), and of employment recovery as a function of policy stringency by gender in panel (b). 95% confidence intervals shown.

We find the relationship between policy stringency and the probability of stopping work over time for a given individual depends on location, gender and education level. Fig. 5(a) shows the probability of stopping work is more strongly associated with policy stringency for urban dwellers compared to those living in rural locations. Women were not significantly more likely to stop work with increases in policy stringency relative to men, but they were less likely to start working during the pandemic (Fig. 5(b)). We find more significant gender differences when considering specific policies within the stringency index. For example, stay-at-home measures had stronger effects on employment loss for women relative to men (Figure A.7). Our results show workers with lower education were less likely to stop working with increasing policy stringency compared to better educated workers (Table 5). This may appear to contradict evidence that more highly educated workers had better chances of working from home. However, studies estimate that only a fraction of jobs in developing countries can be done from home (Gottlieb et al., 2021). Less educated workers are much more likely to work in agriculture or live in rural areas, which were relatively less affected by pandemic restrictions, and some may be too poor to stop working. Our results suggest sectoral and location factors dominated the importance of the ability to work from home in developing country labor markets.

**Table 5:** Effect of policy stringency on employment loss and recovery by population group.

	Stopped working		Started working during pandemic			
			If not working		If stopped working	
	(1)	(2)	(3)	(4)	(5)	(6)
Stringency index	0.033***	0.026***	−0.063***	−0.062***	−0.067***	−0.064***
	(0.002)	(0.006)	(0.003)	(0.010)	(0.005)	(0.017)
Urban × Stringency		0.016***		−0.005		−0.004
		(0.005)		(0.009)		(0.014)
Female × Stringency		0.009		−0.018**		−0.022
		(0.005)		(0.009)		(0.013)
Low education × Stringency		−0.011**		0.022**		0.014
		(0.005)		(0.009)		(0.014)

Respondent FE	✓	✓	✓	✓	✓	✓
R${}^{2}$	0.60	0.62	0.68	0.68	0.64	0.63
N	249,993	148,768	120,927	62,387	70,030	36,673
Countries	51	37	42	30	42	30

*Notes*: The table reports results from a linear probability model with respondent fixed effects. Household sample weights are used within countries and countries are weighted equally. OxCGRT stringency index is matched to surveys at month level and standardized within countries across the period April 2020 to December 2021. Standard errors are robust. * p<0.1, ** p<0.05, *** p<0.01.

COVID-19 High-Frequency Phone Surveys

### 4.3 Employment levels are increasing again, but are the new jobs of the same quality?

The employment gains observed throughout the second half of 2020 and in 2021, albeit incomplete and uneven across countries and population groups, are a welcome sign of recovery. However, simply looking at employment levels may not represent the full picture. Some of the people who regained employment may now hold jobs that are inferior to their previous ones consistent with the widespread income losses documented in the next section. This may be particularly concerning for low-income households, who cannot afford long unemployment spells, especially in the absence of support measures from the government and may need to maintain some means of livelihood to cover basic necessities.

We do not observe job quality directly in the HFPS data, so we rely on several proxies to analyze the quality of net employment changes. In particular, we examine transitions between sectors and types of employment (wage employment vs. self-employment) across different population groups since before the pandemic. In lower-income countries, the agriculture sector oftentimes absorbs workers unable to find work elsewhere during economic shocks. As the economic fallout caused by the pandemic lingers over a longer period, households may rely on agricultural employment as a coping strategy in spite of relatively poor returns. Since we observe the prepandemic sector of employment for each respondent in a subset of surveys, the analysis here relies on the plausible assumption that the movement from non-agricultural employment immediately preceding the COVID-19 pandemic to agricultural employment after the onset of the pandemic is a push factor related to the need to mitigate income losses, rather than a pull factor associated with better remunerated employment in agriculture. Similarly, self-employment which is oftentimes in the informal sector can serve as a buffer against the labor market shock, particularly among households who do not have access to formal or informal safety nets that would help them to wait it out for a better job. As such, it could be argued that transitions from prepandemic wage employment to pandemic self-employment are primarily push factor transitions into immediate, but more precarious and lower-quality employment vis-à-vis the jobs held previously.

HFPS data record a significant share of workers changing jobs during the COVID-19 pandemic. In the early phase of the pandemic, a period also characterized by substantial work stoppages, nine percent of workers reported working in jobs different from their prepandemic employment. Over time, this number rose to more than 20 percent of workers in the second half of 2020. The rate of transitions slowed in 2021, when around one in four workers reported having a different job than before the pandemic. The incidence of job switches is particularly high in LICs (primarily in SSA), with 40 percent of workers reporting having changed jobs by 2021 since the pandemic.

**Fig. 6:**
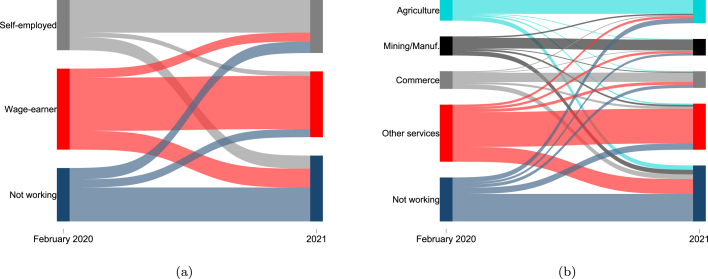
Employment transitions, Prepandemic–2021. *Notes*: The figure shows employment transitions by type of employment in panel (a), and sector of employment in panel (b). The width of each employment category is weighted by prepandemic employment share. The sample for (a) includes 51,911 observed transitions from 38 countries. The sample for (b) includes 57,922 observed transitions from 41 countries. Household sample weights are used within countries and countries are weighted equally. “Other” employment type, accounting for 1 percent of prepandemic employment, is excluded from (a).

We construct transition matrices that track changes in respondent employment type or sector. Because of data limitations, job changes within the same sector or within the same employment type are not captured. For example, the transition matrices do not capture workers who had a job in the services sector in February 2020 and switched jobs within the services sector in 2021, but they would record a movement from services to agriculture, or from wage employment to self-employment. As such, transitions shown in the matrices are lower bounds for the total incidence of job transitions that occurred during this period. Fig. 6 visualizes transitions by employment type and sector between February 2020 (based on recall) and the most recent surveys in 2021. The figure shows the share of the working age population in wage work and in services sectors shrunk significantly, while the share not working grew. Flows from wage work to self-employment were considerably larger than the reverse, and agriculture was the only sector that did not shrink, on average. Full matrices are reported in Tables A.2 and A.3.

There are several notable takeaways from observed transitions. First, the pandemic led to an increase in lower-quality jobs in self-employment. Around eleven percent of prepandemic wage earners transitioned to self-employment, while nine percent of the prepandemic self-employed were wage employees in 2021 (Table A.2). Considering a larger share of the working-age population were wage employees before the pandemic in our sample, this indicates a strong shift of the working-age population into self-employment. In absolute terms, workers were twice as likely to transition from wage employment to self-employment than vice versa. This transition was more prevalent in lower-income countries, with upwards of ten percent of workers entering self-employment.

COVID-19 High-Frequency Phone Surveys.

Second, those self-employed before the pandemic are less likely to be working in 2021 compared to prepandemic wage employees (Table A.2). This is consistent with evidence of greater employment impacts and slower employment gains in sectors such as commerce and other services where self-employment is more prevalent.

Third, existing workers in agriculture were more likely to stay employed than any other sector (Table A.3). It was the only sector that grew, and accounted for 20 percent more of the employed population in 2021, on average, than it did before the pandemic in our sample of 41 countries. The highest incidence of transitions into agriculture occurred in LMICs and LICs. This finding is congruent with earlier evidence that rural areas and agricultural livelihoods were relatively less affected by pandemic-related restrictions and economic fallout in comparison to the urban services sector. Among those who were employed before the pandemic, transitions from mining/manufacturing and commerce into other services sectors were relatively more prevalent than transitions from these two sectors into agriculture, which might reflect both the location and transferable skills of workers most affected.

Finally, the pace of employment gains were particularly slow and inferior transitions were particularly common among women and those with lower levels of education (Tables A.4 and A.5). Among women, 35 percent of prepandemic self-employed and 30 percent of wage workers were not working in 2021 relative to 18 and 19 percent among men, respectively. Women employed in any sector before the pandemic were more likely to be unemployed in 2021 than men from the same sector. Similarly, almost a third of low educated self-employed workers and wage workers before the pandemic were not working in 2021, compared to 19 and 21 percent of higher educated workers starting in the same prepandemic employment category. Low-educated wage workers were three percentage points more likely to transition to self-employment compared to their higher educated peers. Those with lower education in any sector were more likely to transition to agriculture between February 2020 and 2021 than more educated workers in the same sector. Low-educated workers in services sectors were especially more likely to exit the labor market.

## 5 Income dynamics during the pandemic and changes in the intensive margin of employment

This section examines household income dynamics to assess the extent of unrecovered welfare losses throughout the pandemic and relates these losses to employment outcomes using the HFPS data. Outright job losses are but one of the ways in which household welfare was affected. Many workers ended up working fewer hours and receiving smaller pay even if they managed to hold on to their jobs throughout the COVID-19 pandemic. In particular, earnings may be more strongly linked to fluctuations in demand in developing countries, where informal employment is common. As such, many households could have experienced income losses on account of COVID-19 even without any household members losing (or changing) their jobs. Poor households are more exposed to underemployment risks (Golub and Hayat, 2015, Kim and Golden, 2022). Therefore, households may experience unequal welfare impacts not only due to changes at the extensive margin of employment, documented as job loss above, but also in the intensive margin.

Based on HFPS data, income losses were much more widespread than job loss. On average, 65 percent of households reported income loss in the second quarter of 2020, but this share fell over time in most countries (Table A.6). Income losses were prevalent in both farm and nonfarm sectors. Among households that were receiving remittances before the pandemic, half reported losing at least part of that income source during the pandemic. In the second half of 2020, as mobility restrictions started to be relaxed and social assistance reached more households, the share of households reporting a decrease in incomes vis-à-vis prepandemic fell considerably to around 40 percent in MICs but did not decrease in LICs (Table A.6).^14^

Over a quarter of wage workers in LICs and MICs reported losing some of their earnings in the early stages of the pandemic. The same shares were significantly lower in HICs, many of which implemented wage subsidy schemes to support wage workers during the pandemic. Lower earnings among wage workers reflects an adjustment along the intensive margin, such as reduced hours. For example, ILO (2021) estimates 8.8 percent of global working hours were lost in 2020 compared with the fourth quarter of 2019, a loss equivalent to 255 million full-time jobs. About half of the losses are estimated to have come from working hour reductions within employment. The fact many more households reported losing incomes than stopping work, even after accounting for safety net coverage, is consistent with labor markets where many workers are self-employed or hold otherwise informal jobs. Within countries, we find income losses were strongly correlated with stopping work, transitions to self-employment, and transitions to agriculture (Figure A.8).

Given the extent of income losses, the fiscal response to support households appears largely insufficient to offset labor market impacts on household welfare in most LICs and LMICs.^15^ Available data on social protection spending and labor programs confirm large disparities in the amount of support provided to households, with average spending being significantly higher in HICs and MICs than in LICs. Phone surveys show that public assistance appears to have fallen short or was received with a delay, particularly in the poorest countries (World Bank, 2022a).^16^ On average, the share of households that reported having received assistance from the government since the beginning of the pandemic rose from six percent in LICs and 28 percent in UMICs during April-June 2020 to 19 percent and 52 percent respectively in 2021. The difference in social assistance receipt across population groups tends to be relatively small. Rural households were slightly more likely to benefit from assistance, though the difference with urban households remained small even later in the pandemic. Households where respondents had lower education levels were also more likely to receive assistance. Households with children were more likely to receive government assistance than households without any children, initially and also later in the pandemic (World Bank & UNICEF, 2022).^17^

Evidence of uneven welfare impacts across population groups is made clear by comparing characteristics associated with income loss and recovery in multivariate regressions. We follow the linear probability model in specification (1), but with income loss and recovery outcomes. Similar to analysis of employment recovery, we employ panel surveys in about 20 countries that collected data on income changes between survey waves within the pandemic, as well as capturing initial income losses relative to before the pandemic in early survey rounds. We can then identify which households initially experiencing income loss were more likely to recover at least some income, as reported in a subsequent HFPS wave.

**Fig. 7:**
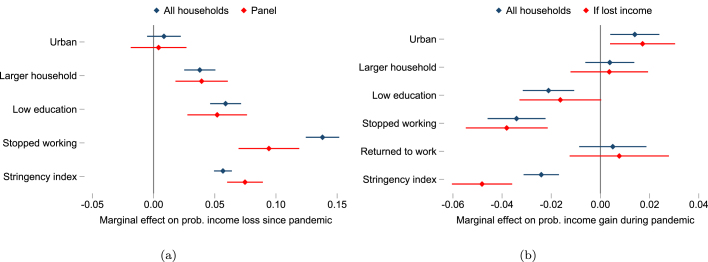
Characteristics associated with income loss and recovery. *Notes*: The figure visualizes results in Table 6. It shows the marginal effect of characteristics on the probability of income loss relative to the prepandemic reference period in panel (a), and of income gain during the pandemic in panel (b). 95% confidence intervals shown.

Regression results are reported in Table 6 and shown in Fig. 7.^18^ Fig. 7(a) shows that within countries, households larger than the country median, households where the respondent had lower education or had stopped working, and periods of more stringent policies are each associated with a higher probability of income loss. Fig. 7(b) shows that some of the same households were less likely to subsequently gain income during the pandemic. Households where respondents returned to work were five percentage points more likely to report income gains during the pandemic compared to those who had not recovered employment. By implication, the same groups less likely to recover employment were also less likely to recover income (Fig. 7(b)). Results are consistent with income losses in the intensive margin of employment. For instance, controlling for changes in employment at the extensive margin, we find that households with less access to education were both more likely to lose income relative to prepandemic and less likely to gain or recover income during the pandemic era. Urban households were slightly more likely to recover income than rural households. Initial income losses were higher when policy stringency was higher. Rates of recovery were also lower when policy stringency was higher, even controlling for household characteristics and employment status. We observe a similar relationship between policy stringency and the probability of income loss and recovery for a given household when using the panel model in specification (2) that controls for all time-invariant characteristics at household level (Table A.6).^19^

**Table 6:** Characteristics associated with income loss and recovery during the pandemic.

	Income loss		Income gain during pandemic	
	All households	Panel	All households	If lost income
	(1)	(2)	(3)	(4)
Urban	0.008	0.004	0.014***	0.017**
	(0.007)	(0.012)	(0.005)	(0.007)
Larger household	0.038***	0.039***	0.004	0.004
	(0.006)	(0.011)	(0.005)	(0.008)
Low education	0.059***	0.052***	−0.021***	−0.016*
	(0.006)	(0.012)	(0.005)	(0.008)
Stopped working	0.138***	0.094***	−0.034***	−0.038***
	(0.007)	(0.013)	(0.006)	(0.008)
Returned to work			0.005	0.008
			(0.007)	(0.010)
Stringency index	0.057***	0.075***	−0.024***	−0.048***
	(0.004)	(0.007)	(0.004)	(0.006)

Country FE	✓	✓	✓	✓
R${}^{2}$	0.13	0.12	0.05	0.03
N	71,954	21,174	44,459	19,119
Countries	41	19	22	17

*Notes*: The table reports results from a linear probability model with country fixed effects. Household sample weights are used within countries and countries are weighted equally. The OxCGRT stringency index is matched to surveys at month level and standardized within countries across the period April 2020 to December 2021. Standard errors are robust. * p<0.1, ** p<0.05, *** p<0.01.

COVID-19 High-Frequency Phone Surveys.

## 6 Concluding remarks

This study analyzed the welfare impacts of the COVID-19 pandemic using a harmonized database of the World Bank’s high-frequency phone surveys in 80 countries over 2020 and 2021 — the largest source of household level data from developing countries during the pandemic. Focusing on the scarring effects of the pandemic, we show that, while there have been some improvements in employment levels, the labor market impacts continue to be felt years into the pandemic. Employment dynamics suggest that initial disparities observed early in the COVID-19 pandemic narrowed rather slowly over time and sometimes not at all. Women and less educated workers continue to report a higher incidence of work stoppages into 2021 compared to men and better educated groups, respectively. Using panel regressions, we show the relationship between policy stringency and employment outcomes over time for a given worker depends on location, gender and education level.

Our study also uncovers broader labor market impacts, beyond adjustments at the extensive margin. Analysis of job transitions throughout the pandemic shows substantial churn in the labor market, including significant shifts from wage employment to self-employment and from non-agriculture sectors to agriculture, likely reflecting a rise in informal and more precarious employment accompanied with lower returns. Lower socio-economic groups, as proxied by the respondent’s level of education, were more likely to transition into lower-quality jobs, while also being less likely to recover lost employment. Observed household income dynamics are consistent with reported job loss and labor market transitions. They point to compounding unequal welfare impacts due to changes in the intensive margin of employment, particularly for households with less educated respondents, and indicate pandemic era public assistance was insufficient to fully compensate losses.

In conclusion, unrecovered and unmitigated welfare losses from the COVID-19 crisis due to labor market impacts could worsen inequality in the longer term, leaving “long COVID” scars on a post-pandemic economy. Job and income losses persisted especially among poorer population groups and countries. The adoption of negative coping strategies, including the use of savings and sale of assets, could further hurt households’ productive capacity and ability to recover from the crisis. Combined with the impact of unequal learning losses, the full impact of the crisis may only be seen over the long term. One may ask, at the same time, whether pre-existing inequalities have contributed to more unequal pandemic impacts, consistent with a negative feedback loop (Hill & Narayan, 2020). This is an important question for future research when reliable post pandemic income distribution data becomes available.

## CRediT authorship contribution statement

**Ben Brunckhorst:** Conceptualization, Data curation, Formal analysis, Writing – original draft, Writing – review & editing, Visualization. **Alexandru Cojocaru:** Conceptualization, Writing – original draft, Writing – review & editing. **Yeon Soo Kim:** Conceptualization, Data curation, Writing – original draft, Writing – review & editing, Supervision. **Maurice Kugler:** Writing – original draft.

## Declaration of competing interest

The authors have no conflicts of interest.

## Data Availability

The authors do not have permission to share data.

## Appendix A Supplementary data

MMC S1 Supplementary data and analysis.

## Footnotes

1 For comparison, the analysis of initial impacts by Egger et al. (2021) used data from nine developing countries, and Bundervoet et al. (2022) used representative phone surveys from 31 countries. While phone surveys were conducted in eight countries in South Asia, the region is excluded from the analysis because it was not possible to harmonize employment indicators due to differences in the survey instrument.

2 The grouping of countries follows the World Bank country classifications by income level for FY21: https://datahelpdesk.worldbank.org/knowledgebase/articles/906519.

3 There is a substantive literature documenting the benefits of large-scale anti-contagion policies that is beyond the scope and focus of this paper.

4 Evidence is more abundant in high-income countries. Sostero et al. (2020) find that in Europe three-quarters of employees in the top wage quintile were able to work remotely, compared to only 3 percent of those in the bottom quintile. Adams-Prassl et al. (2022) similarly find that in the UK workers with gross labor income above GBP 70,000 can accomplish some 60 percent of tasks remotely, compared to 20 percent among workers with gross labor income below GBP 10,000.

5 While traditionally vulnerable groups appear to have experienced greater losses as seen in group-wise comparisons, the final impact on within-country inequality depends on where these groups are located in the overall income distribution. Mahler et al. (2022) estimate the change in the Gini index by combining prepandemic household survey data with information from high-frequency phone surveys and macroeconomic growth projections. These estimates are combined with data from NSOs, household surveys conducted in 2020 and estimates from the literature. Results show a mix of positive and negative changes in within-country inequality, with the magnitude being small in most cases.

6 Figure A.1 lists all countries with HFPS data included in the analysis and shows the timing of surveys at country level.

7 Individual level sampling weights are only available in some countries with RDD surveys, and it would be challenging to use these for cross-country comparisons with countries without individual level weights.

8 The OxCGRT stringency index records the strictness of “lockdown style” policies that primarily restrict people’s behavior. It is calculated using 9 indicators including school closures; workplace closures; cancellation of public events; restrictions on public gatherings; closures of public transport; stay-at-home requirements; public information campaigns; restrictions on internal movements; and international travel controls (Hale et al., 2021).

9 The main findings from the descriptive analysis are consistent if we instead pool surveys within each period. The regression analysis does not compare across time periods, but rather pools all HFPS survey rounds, weighting countries equally.

10 For example, the HPFS question asking whether a respondent worked before the COVID-19 pandemic could be capturing broader recall of past employment which may or may not be coded as employment in a considerably more detailed LFS instrument. Respondents reporting not working in the HFPS after the onset of COVID-19 could still be classified as employed in the LFS as they are temporarily absent from work but believe they still have a job to go back to in the future. Differences in the magnitude of employment impacts are in line with expectations considering these factors. Also, the LFS available for comparison are conducted quarterly which does not line up with the timing of HFPS and therefore may not capture the same short-term disruptions to the labor market.

11 In some surveys, prepandemic employment variables were collected only for those not working at the time of the first survey wave. When questions were skipped for those working, we assume prepandemic employment variables (status, sector and employment type) are the same as those reported in the first survey round. We find these assumptions hold true in 93–96 percent of cases where prepandemic data was collected for all respondents in the first wave (N = 51,186). Similar assumptions were made in previous analysis (Bundervoet et al., 2022), but we validate them for the first time. Details are available from the authors.

12 One method to assess potential recall bias in prepandemic employment estimates is to observe survey-to-survey recall bias when HFPS included retrospective questions about employment in the previous round. We find recall based employment estimates were not significantly different between survey rounds. An alternate method compares recall-based estimates from HFPS with estimates from LFS data collected shortly before the pandemic. We find prepandemic employment levels in the HFPS to be six percentage points higher, on average, than those using LFS data (26 countries). However, this deviation captures not only potential recall bias but also the effect of differences in sampling, timing and question framing between the data sources. For example, Kugler et al. (2023) find employment levels tend to be overstated in HFPS on account of respondent selection, and LFS estimates use a narrower definition of employment than the phone surveys. Nonetheless, employment losses persisting in 2021 remain larger than these upper bound estimates of recall bias based on LFS for 16 of 21 countries with both data sources, implying evidence of unrecovered losses is robust.

13 Results are robust to alternate definitions of the outcomes or logit and probit model specifications.

14 The sample of LICs with income loss data is small, and it is difficult to construct a balanced country panel since fewer surveys collected comparable data on changes in household income.

15 Sudden and widespread losses of livelihoods at the beginning of the pandemic led to a fiscal response of unprecedented scale around the world. According to the IMF COVID-19 fiscal policies database, countries have collectively spent over $13 trillion on mitigation measures since the beginning of the pandemic (IMF, 2021). Nearly 4,000 social protection and labor measures were planned or implemented in 223 economies as of January 2022 (Gentilini et al., 2022). However, the magnitude and composition of the fiscal package varied across countries, with HICs on average spending significantly more, particularly on support toward households and firms (Benmelech and Tzur-Ilan, 2020, IMF, 2021).

16 Phone survey data are an imperfect source for assessing social assistance coverage during the pandemic. This is because the survey collected only a binary variable indicating whether assistance was received, and often times no distinction was made between assistance from pre-existing programs or new COVID-19-related initiatives. The wording of the questions was such that it usually included cash and in-kind transfers, but was less likely to include support received in the form of subsidies or tax/payment exemptions. The timing of the survey does not precisely capture the timing of support. Nevertheless, the HFPS are a rare source of cross-country information on the extent to which some form of fiscal support reached households at different times over the pandemic.

17 The challenges with targeting are in part due to existing constraints of the social protection system in poorer countries, in particular high levels of informality and the lack of a social registry which could be used to quickly reach those in need. In fact, cash transfers, the main form of income support provided during the pandemic, were directed at informal workers in 79 percent of programs. In contrast, wage subsidies which mainly benefit formal workers were the most common labor market policy directed at individuals in HICs (Kamran et al., 2023). Many governments relied on inefficient subsidies instead of targeted transfers to respond to the crisis (World Bank, 2022a).

18 Results are robust to alternate definitions of the outcomes or specifications of the model (logit, probit).

19 We do not find significant heterogeneity in terms of the effect of policy stringency on income loss and recovery by location, household size or education in the panel household model. The panel household sample is much more limited for income outcomes compared to employment outcomes, with only 7 countries collecting data on income gain across multiple survey rounds during the pandemic. Results are reported in Table A.7.
