# Supplementary material for: Long COVID: The evolution of household welfare in developing countries during the pandemic
Source: World Dev. 2024 Mar;175:106485. doi: 10.1016/j.worlddev.2023.106485 (PMC11639124; doi:10.1016/j.worlddev.2023.106485)
Supplement: MMC S1 — Supplementary data and analysis. [file mmc1.pdf]

# Appendix A. Supplementary material for *Long Covid: The Evolution of Household Welfare in Developing Countries during the Pandemic*

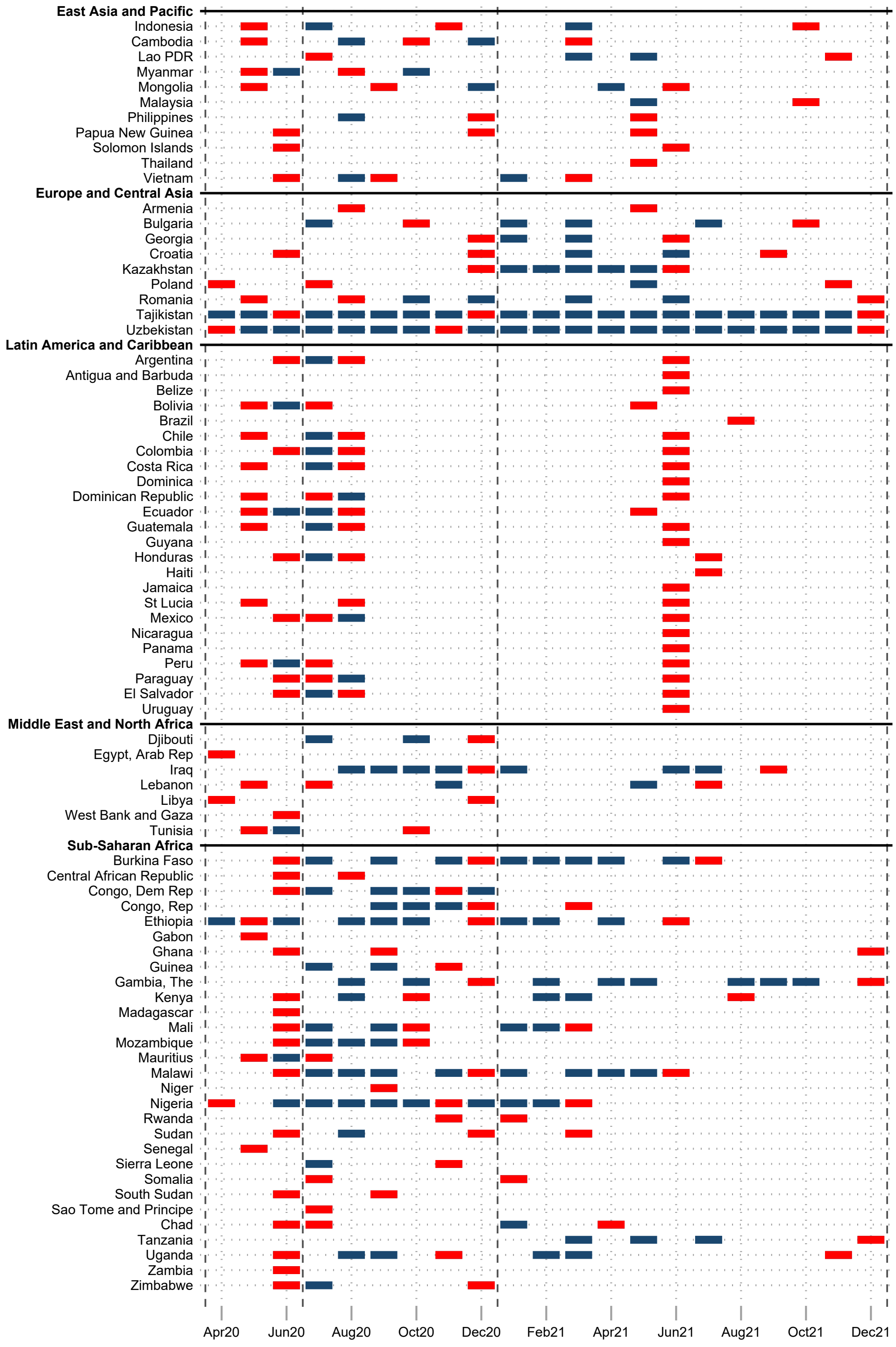


Figure A.1: Timing of HFPS waves by country, sorted by region



*Notes*: Surveys used in analysis by time period are shown in red. Surveys were attributed to the mean month of data collection.

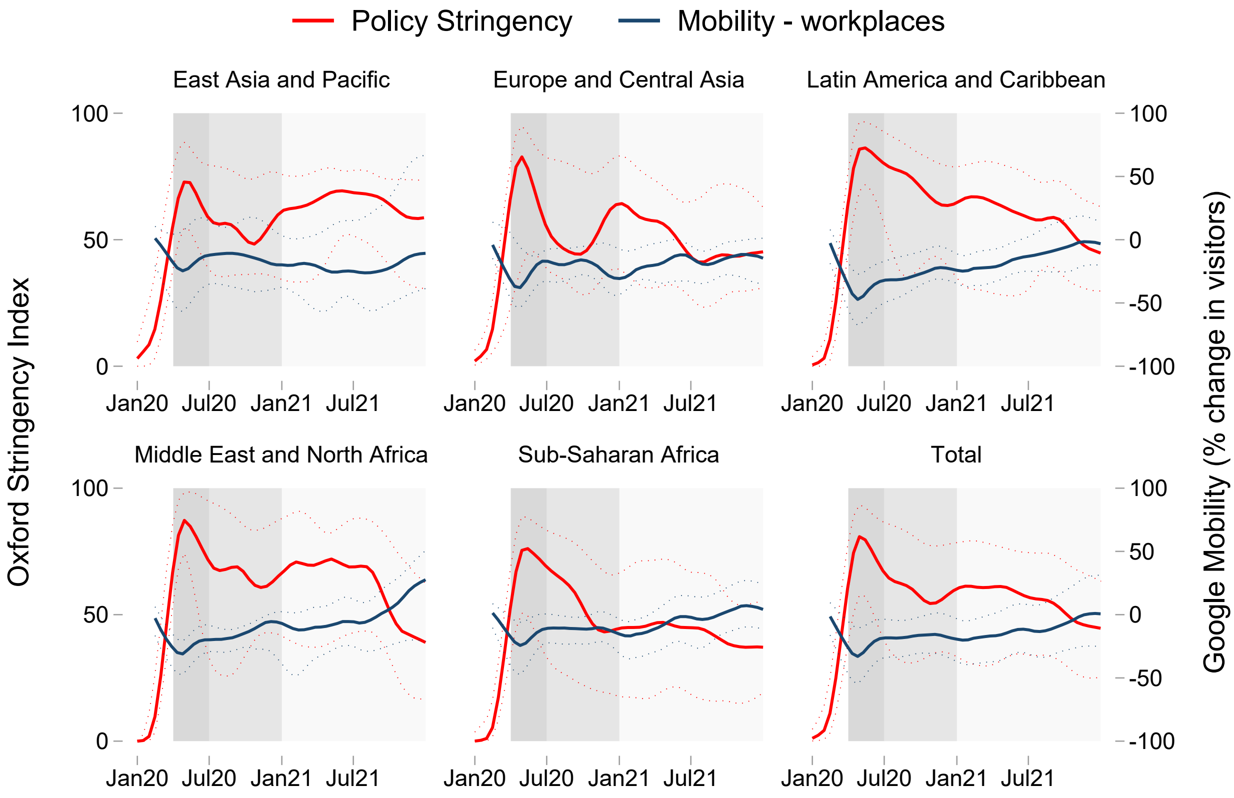


Figure A.2: Policy stringency and mobility trends over time in countries included in analysis, by region

*Source*: Google COVID-19 Community Mobility Reports and Oxford COVID-19 Government Response Tracker.
*Notes*: The shaded regions represent the three time periods used for descriptive analysis. Solid lines indicate the median of each indicator and dotted lines show the 10th and 90th percentiles. The OxCGRT stringency index is available for 76 out of 80 countries with HFPS data. Google Community Mobility data is available for 57 countries with HFPS data.

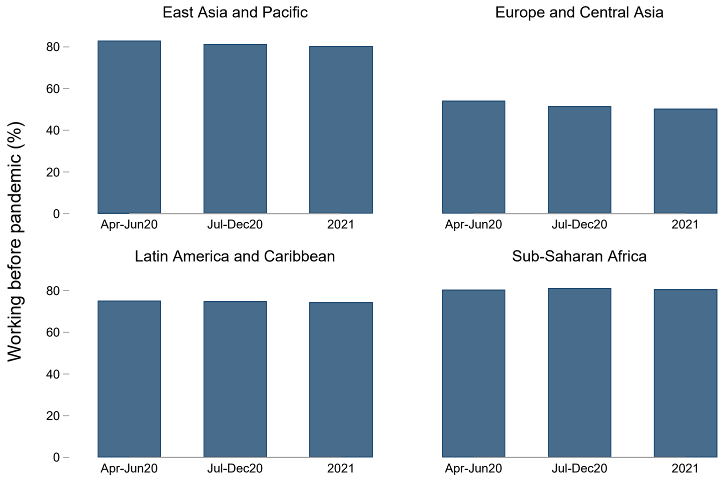


Figure A.3: Prepandemic employment estimates over time

*Source*: COVID-19 High-Frequency Phone Surveys.

*Notes*: The sample includes 31 countries with prepandemic employment estimates from HFPS in all three periods (EAP: 5, ECA: 4, LAC: 14, SSA: 8).

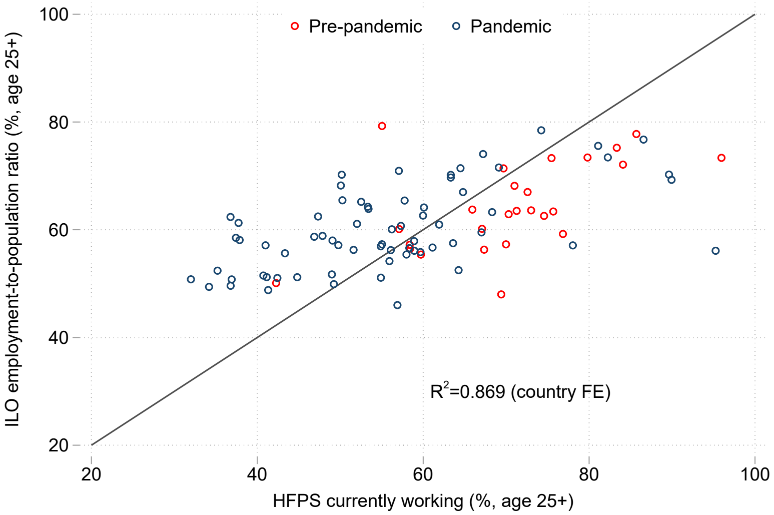


Figure A.4: Correlation between ILO and HFPS employment estimates

*Source*: COVID-19 High-Frequency Phone Surveys and ILOSTAT.

*Notes*: The sample includes 94 estimates from 28 economies. HFPS data are matched to ILO data at quarter level. Sources of error include differences in sampling, timing, and instrument design, including potential recall bias for the HFPS prepandemic baseline.

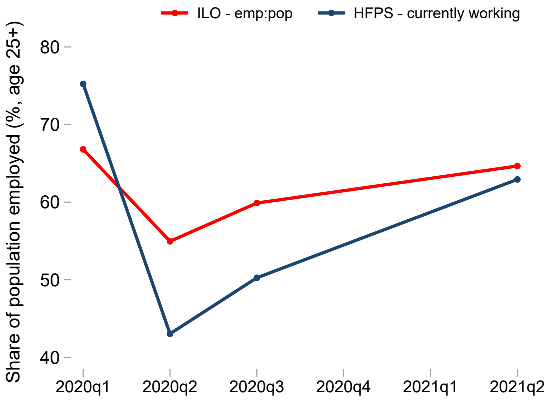


(a) ILO vs HFPS currently working

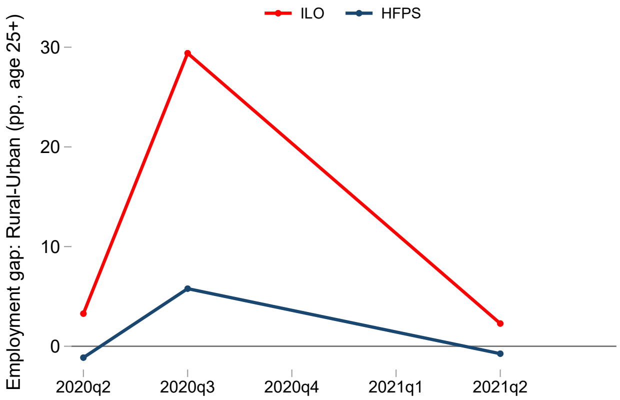


(b) Rural/Urban gap

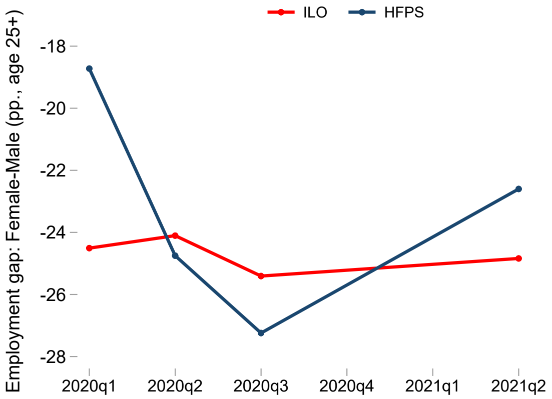


(c) Gender gap

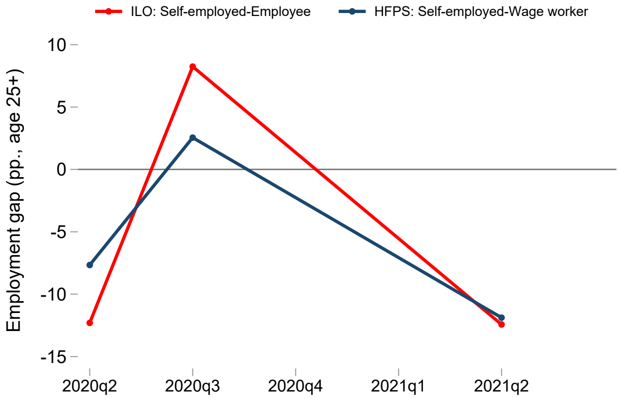


(d) Employment type gap

Figure A.5: ILO and HFPS employment estimates over time in LAC

*Source*: COVID-19 High-Frequency Phone Surveys and ILOSTAT.

*Notes*: LAC aggregate for 9-11 countries with data collected in the same quarter.

Table A.1: Relative employment losses over time

| | Employment loss relative to prepandemic level (%) | | | Sample | |
|---|---|---|---|---|---|
| | **Apr-Jun20** | **Jul-Dec20** | **2021** | **Countries** | **N** |
| All countries | 30.6 | 16.8 | 8.2 | 75 | 246,618 |
| HICs | 18.0 | 17.9 | 15.3 | 8 | 18,740 |
| UMICs | 38.6 | 22.1 | 7.7 | 24 | 74,561 |
| LMICs | 33.8 | 16.0 | 4.6 | 25 | 92,293 |
| LICs | 23.2 | 11.8 | 9.8 | 18 | 61,024 |
| East Asia and Pacific | 20.2 | 12.0 | 7.3 | 11 | 54,933 |
| Europe and Central Asia | 22.3 | 10.3 | 0.5 | 8 | 32,724 |
| Latin America and Caribbean | 44.5 | 31.0 | 14.8 | 24 | 52,550 |
| Middle East and North Africa | 37.3 | 7.2 | 6.6 | 6 | 21,441 |
| Sub-Saharan Africa | 24.3 | 13.3 | 0.3 | 26 | 84,970 |
| **Sample restricted to a country panel** | | | | | |
| All countries | 31.0 | 19.2 | 12.0 | 31 | 140,798 |
| HICs | 17.9 | 17.9 | 14.2 | 4 | 15,719 |
| UMICs | 38.6 | 27.7 | 15.0 | 12 | 48,113 |
| LMICs | 36.3 | 19.5 | 11.3 | 9 | 41,511 |
| LICs | 16.6 | 2.7 | 5.6 | 6 | 35,455 |
| East Asia and Pacific | 15.2 | 15.8 | 11.1 | 5 | 36,142 |
| Europe and Central Asia | 22.3 | 11.6 | 15.6 | 4 | 16,735 |
| Latin America and Caribbean | 44.5 | 31.0 | 14.6 | 14 | 41,119 |
| Middle East and North Africa | 24.3 | 17.2 | 10.0 | 1 | 5,991 |
| Sub-Saharan Africa | 21.3 | 2.6 | 5.6 | 7 | 40,811 |
| Female | 35.8 | 22.6 | 13.0 | 31 | 60,950 |
| Male | 27.7 | 16.1 | 10.4 | 31 | 79,353 |
| **Female/Male** | 1.29 | 1.40 | 1.25 | | |
| Rural | 28.4 | 16.6 | 11.6 | 27 | 53,174 |
| Urban | 31.3 | 20.0 | 14.2 | 27 | 73,999 |
| **Rural/Urban** | 0.91 | 0.83 | 0.82 | | |
| Low education | 35.2 | 22.5 | 12.6 | 23 | 55,169 |
| High education | 28.2 | 20.7 | 12.7 | 23 | 47,497 |
| **Low/High** | 1.25 | 1.09 | 0.99 | | |

*Source*: COVID-19 High-Frequency Phone Surveys.

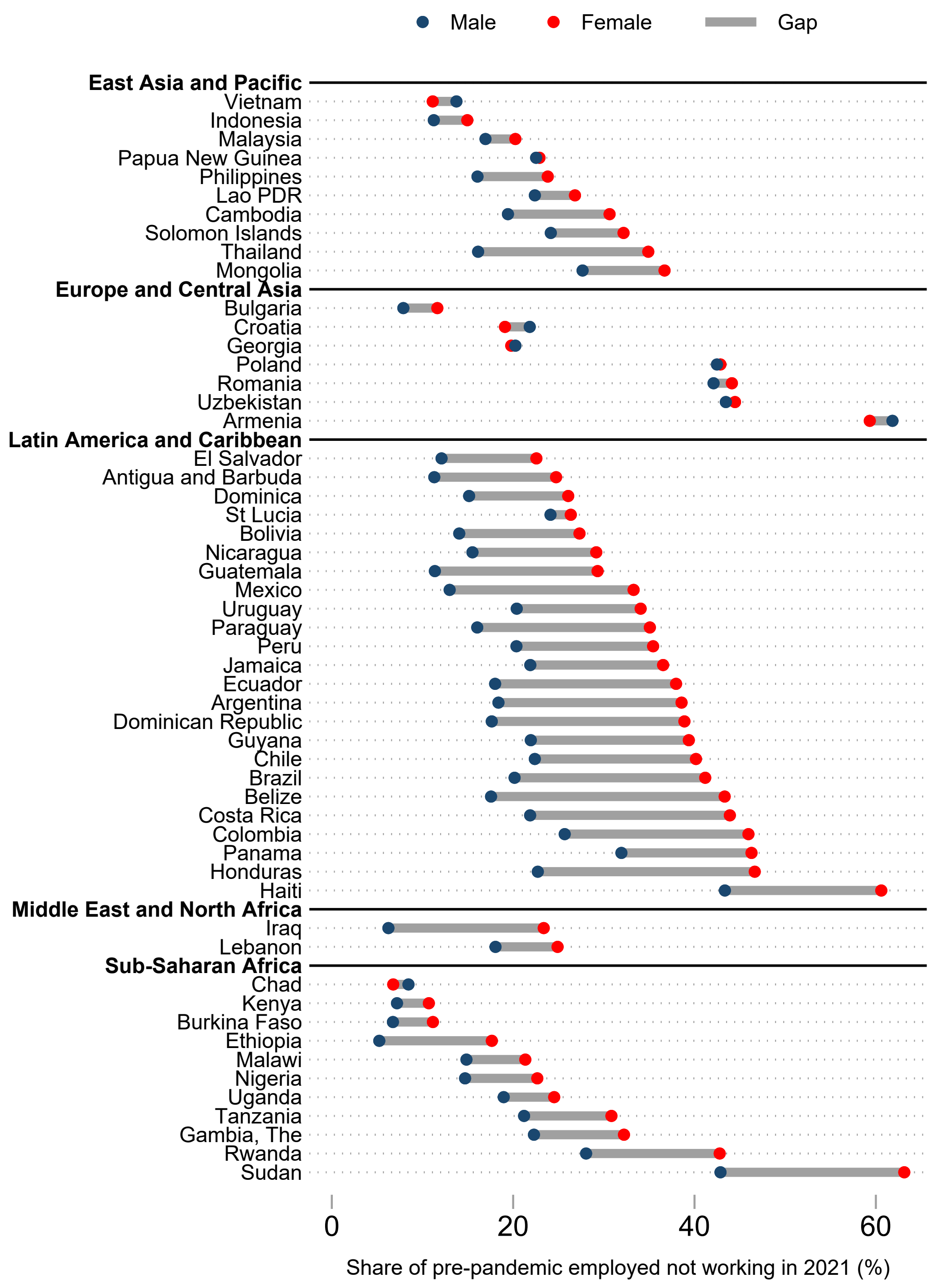


Figure A.6: Employment loss in 2021 by gender and country

*Source*: COVID-19 High-Frequency Phone Surveys.

*Notes*: The figure shows the share of those working before the pandemic who were not working in 2021, by gender. Household sample weights are used within countries.

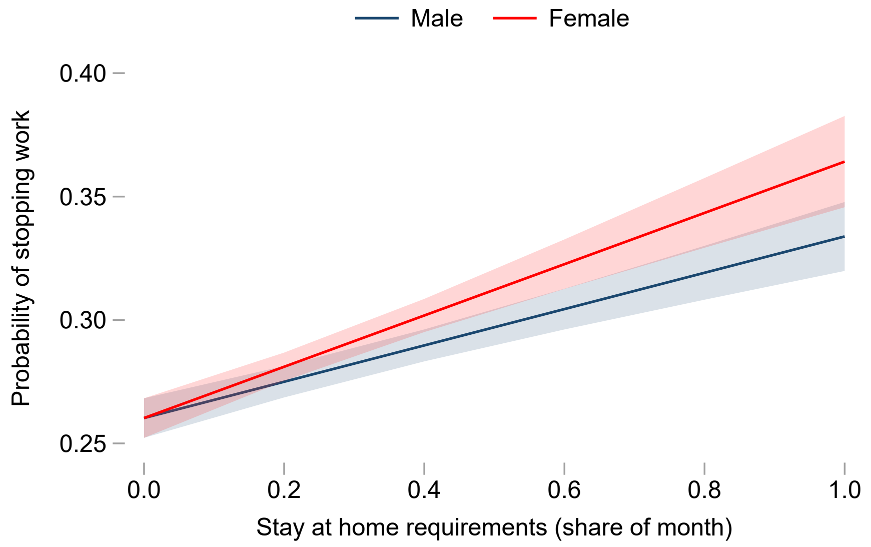


Figure A.7: Effect of stay-at-home measures on employment loss, by gender

*Source*: COVID-19 High-Frequency Phone Surveys.

Table A.2: Employment type transition matrix, February 2020–2021

| | **Feb20 share (%)** | **Share of Feb20 type by 2021 type (%)** | | | | **2021 share (%)** |
|---|---|---|---|---|---|---|
| | | Self | Wage | Other | Not work | |
| **Self-employed** | 27 | **65** | 9 | 0 | 26 | 28 |
| **Wage-earner** | 44 | 11 | **65** | 0 | 23 | 35 |
| **Other** | 1 | 20 | 21 | **30** | 30 | 1 |
| **Not working** | 29 | 21 | 15 | 1 | **63** | 36 |

*Source*: COVID-19 High-Frequency Phone Surveys.

*Notes*: Row percentages across 2021 employment types add to 100 for each prepandemic employment type. The sample includes 51,911 observed transitions from 38 countries (4 HICs, 23 UMICs, 7 LMICs, and 4 LICs). Household sample weights are used within countries and countries are weighted equally.

Table A.3: Employment sector transition matrix, February 2020–2021

| | **Feb20 share (%)** | **Share of Feb20 sector by 2021 sector (%)** | | | | | **2021 share (%)** |
|---|---|---|---|---|---|---|---|
| | | Agric. | Min./Manuf. | Comm. | Other | Not work | |
| **Agriculture** | 13 | **67** | 3 | 3 | 6 | 21 | 14 |
| **Mining/Manuf.** | 12 | 7 | **56** | 4 | 9 | 24 | 11 |
| **Commerce** | 11 | 5 | 4 | **53** | 12 | 27 | 10 |
| **Other services** | 36 | 4 | 4 | 5 | **61** | 26 | 29 |
| **Not working** | 28 | 11 | 5 | 7 | 14 | **62** | 36 |

*Source*: COVID-19 High-Frequency Phone Surveys.

*Notes*: Row percentages across 2021 employment types add to 100 for each prepandemic employment sector. The sample includes 57,922 observed transitions from 41 countries (4 HICs, 21 UMICs, 10 LMICs, and 6 LICs). Household sample weights are used within countries and countries are weighted equally.

Table A.4: Employment type transitions by education level and gender, February 2020–2021

| | **Education level** | **Feb20 share (%)** | **Share of Feb20 type by 2021 type (%)** | | | | **2021 share (%)** |
|---|---|---|---|---|---|---|---|
| | | | Self | Wage | Other | Not work | |
| **Self-employed** | Low | 28 | **62** | 8 | 0 | 29 | 28 |
| | High | 21 | **70** | 9 | 0 | 21 | 22 |
| **Wage-earner** | Low | 39 | 11 | **60** | 0 | 29 | 30 |
| | High | 55 | 8 | **72** | 0 | 19 | 48 |
| **Other** | Low | 1 | 18 | 21 | **32** | 29 | 1 |
| | High | 1 | 16 | 20 | **31** | 32 | 1 |
| **Not working** | Low | 32 | 20 | 13 | 1 | **67** | 41 |
| | High | 23 | 18 | 21 | 1 | **61** | 29 |
| | **Gender** | **Feb20 share (%)** | **Share of Feb20 type by 2021 type (%)** | | | | **2021 share (%)** |
| | | | Self | Wage | Other | Not work | |
| **Self-employed** | Male | 30 | **71** | 10 | 0 | 19 | 32 |
| | Female | 23 | **57** | 8 | 0 | 35 | 25 |
| **Wage-earner** | Male | 49 | 11 | **70** | 1 | 18 | 42 |
| | Female | 39 | 12 | **58** | 0 | 30 | 29 |
| **Other** | Male | 1 | 16 | 24 | **39** | 20 | 1 |
| | Female | 1 | 20 | 20 | **19** | 41 | 1 |
| **Not working** | Male | 19 | 25 | 22 | 1 | **52** | 25 |
| | Female | 37 | 19 | 12 | 1 | **68** | 45 |

*Source*: COVID-19 High-Frequency Phone Surveys.

*Notes*: Row percentages across 2021 employment types add to 100 for each prepandemic employment type. The sample includes 46,094 observed transitions from 34 countries by education level, and 51,759 observed transitions from 38 countries by gender. Household sample weights are used within countries and countries are weighted equally.

Table A.5: Employment sector transitions by education level and gender, February 2020–2021

| | Education level | Feb20 share (%) | Share of Feb20 sector by 2021 sector (%) | | | | | 2021 share (%) |
|---|---|---|---|---|---|---|---|---|
| | | | Agric. | Min./Manuf. | Comm. | Other | Not work | |
| **Agriculture** | Low | 14 | **68** | 3 | 3 | 6 | 21 | 16 |
| | High | 6 | **72** | 3 | 2 | 7 | 16 | 7 |
| **Mining/Manuf.** | Low | 13 | 6 | **54** | 3 | 12 | 25 | 11 |
| | High | 11 | 3 | **61** | 3 | 8 | 24 | 10 |
| **Commerce** | Low | 11 | 4 | 4 | **49** | 9 | 33 | 9 |
| | High | 11 | 2 | 3 | **61** | 10 | 24 | 11 |
| **Other services** | Low | 30 | 4 | 4 | 4 | **56** | 32 | 23 |
| | High | 48 | 2 | 3 | 4 | **69** | 21 | 41 |
| **Not working** | Low | 32 | 11 | 5 | 7 | 12 | **65** | 41 |
| | High | 24 | 7 | 5 | 8 | 20 | **60** | 31 |

| | Gender | Feb20 share (%) | Share of Feb20 sector by 2021 sector (%) | | | | | 2021 share (%) |
|---|---|---|---|---|---|---|---|---|
| | | | Agric. | Min./Manuf. | Comm. | Other | Not work | |
| **Agriculture** | Male | 16 | **72** | 3 | 3 | 6 | 17 | 18 |
| | Female | 9 | **57** | 2 | 3 | 6 | 31 | 10 |
| **Mining/Manuf.** | Male | 18 | 8 | **59** | 3 | 9 | 21 | 16 |
| | Female | 6 | 5 | **47** | 5 | 10 | 33 | 5 |
| **Commerce** | Male | 10 | 6 | 6 | **58** | 13 | 17 | 9 |
| | Female | 12 | 4 | 2 | **49** | 11 | 35 | 12 |
| **Other services** | Male | 36 | 5 | 5 | 4 | **66** | 19 | 30 |
| | Female | 36 | 4 | 2 | 6 | **56** | 32 | 27 |
| **Not working** | Male | 20 | 16 | 9 | 6 | 17 | **52** | 26 |
| | Female | 36 | 8 | 3 | 8 | 12 | **68** | 45 |

*Source*: COVID-19 High-Frequency Phone Surveys.

*Notes*: Row percentages across 2021 employment types add to 100 for each prepandemic employment sector. The sample includes 52,387 observed transitions from 37 countries by education level, and 57,770 observed transitions from 41 countries by gender. Household sample weights are used within countries and countries are weighted equally.

Table A.6: Total income decreased since pandemic (share of households)

| | Household income decreased since pandemic (%) | | | Sample | |
|---|---|---|---|---|---|
| | **Apr-Jun20** | **Jul-Dec20** | **2021** | **Countries** | **N** |
| All countries | 64.7 | 42.8 | 48.1 | 51 | 120,349 |
| HICs | 42.4 | 28.9 | 38.9 | 8 | 13,972 |
| UMICs | 65.0 | 35.8 | 50.6 | 22 | 44,138 |
| LMICs | 71.8 | 38.7 | 44.2 | 16 | 47,787 |
| LICs | 67.6 | 67.0 | 69.8 | 5 | 14,452 |
| East Asia and Pacific | 76.4 | 37.7 | 45.5 | 8 | 20,561 |
| Europe and Central Asia | 46.0 | 33.7 | 25.5 | 8 | 24,665 |
| Latin America and Caribbean | 65.5 | - | 52.8 | 24 | 41,922 |
| Middle East and North Africa | 62.6 | 43.8 | - | 2 | 10,271 |
| Sub-Saharan Africa | 68.2 | 67.0 | 67.2 | 9 | 22,930 |
| Rural | 67.0 | 38.7 | 48.7 | 49 | 43,755 |
| Urban | 64.2 | 40.3 | 47.4 | 49 | 70,399 |
| Low education | 63.6 | 45.9 | 49.6 | 44 | 47,210 |
| High education | 58.3 | 42.3 | 45.2 | 44 | 44,778 |

*Source*: COVID-19 High-Frequency Phone Surveys.

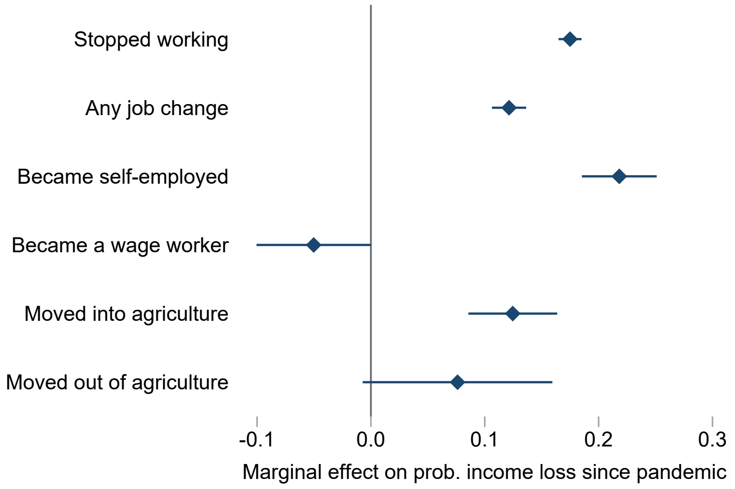


Figure A.8: Relationship between employment transitions and income loss within countries

*Source*: COVID-19 High-Frequency Phone Surveys.

*Notes*: The table reports results from bivariate regressions with country fixed effects. The sample pools all HFPS waves with response rates of at least 50 percent for the variables in the regression. Household sample weights are used within countries and countries are weighted equally. Sample size ranges from 22,026 respondents in 24 countries (employment type transitions) to 162,885 respondents in 49 countries (stopped working). Standard errors are robust. 95% confidence intervals shown.

Table A.7: Effect of policy stringency on income loss and recovery by population group

| | Income loss | | Income gain during pandemic | | | |
|---|---|---|---|---|---|---|
| | | | All households | | If lost income | |
| | (1) | (2) | (3) | (4) | (5) | (6) |
| Stringency index | 0.036*** | 0.043*** | −0.013*** | −0.035*** | −0.017*** | −0.032*** |
| | (0.002) | (0.006) | (0.002) | (0.005) | (0.003) | (0.009) |
| Urban × Stringency | | 0.009 | | 0.025*** | | 0.015 |
| | | (0.006) | | (0.006) | | (0.010) |
| Larger household × Stringency | | 0.003 | | 0.011* | | −0.009 |
| | | (0.006) | | (0.006) | | (0.010) |
| Low education × Stringency | | 0.006 | | 0.011* | | 0.011 |
| | | (0.006) | | (0.006) | | (0.009) |
| Household FE | ✓ | ✓ | ✓ | ✓ | ✓ | ✓ |
| $R^2$ | 0.58 | 0.59 | 0.39 | 0.43 | 0.39 | 0.45 |
| N | 153,415 | 99,647 | 77,776 | 41,713 | 45,519 | 18,096 |
| Countries | 31 | 27 | 9 | 7 | 7 | 5 |

*Source*: COVID-19 High-Frequency Phone Surveys.

*Notes*: The table reports results from a linear probability model with household fixed effects. Household sample weights are used within countries and countries are weighted equally. OxCGRT stringency index is matched to surveys at month level and standardized within countries across the period April 2020 to December 2021. Standard errors are robust. * $p < 0.1$, ** $p < 0.05$, *** $p < 0.01$.
